# New generation of highly sensitive radon detectors based on activated carbon with compensated temperature dependence

**DOI:** 10.1038/s41598-022-12502-z

**Published:** 2022-05-19

**Authors:** Dobromir Pressyanov

**Affiliations:** Faculty of Physics, University of Sofia “St. Kliment Ohridski”, Sofia, Bulgaria

**Keywords:** Environmental sciences, Characterization and analytical techniques, Risk factors

## Abstract

Recently patented compensation module for sensors of radioactive noble gases paves the way for novel designs of radon detectors/monitors with adsorbents, suitable for long-term measurements. The module compensates the strong influence of the temperature on the response of detectors with activated carbon or other ad/absorbents. This report describes radon detectors in which activated carbon fabric is coupled with a solid state nuclear track detector and placed in a compensation module. The module is a hermetic volume made of polyethylene foil, through which the radon diffuses. Two reciprocal trends make possible the temperature compensation: that of the radon penetration through the walls, which increases with the increase of the temperature, and that of the adsorption, which decreases. The results show that outside the module the variation of the detector response over the range of 5–35 °C is 230–305%. In contrast, inside the module the variation is reduced to 6–17%. The module also protects the sensor from humidity and thoron, keeping its sensitivity to radon 7–9 times higher than that of commonly used radon detectors. This makes the new detectors very useful for measurement of low radon concentrations in the atmosphere.

Based on direct epidemiological evidence, exposure to radon (^222^Rn, half-life 3.82 d) is recognized as the second cause of lung cancer, after smoking and the reason number one for never smokers^1^. In result many countries upgraded their radiation protection legislation to incorporate radon exposure in a proper way. In particular, in European union (EU) a reference level for the annual average radon activity concentration was recommended. The reference level in the EU member states should not exceed 300 Bq m^−3^, both for dwellings and working places^2^. In the last years interest in the monitoring of low radon concentrations, typical for the outdoor air also arose^3^. Besides for radiation environment research, such monitoring is important for the evaluation of the atmospheric transport models used to assess the greenhouse gases inventory, in the light of the United Nations Framework Convention and Paris Agreement on Climate Change. Moreover, it is planned to include sensitive radon detectors/monitors in climate monitoring networks. In this respect research efforts were reinforced to develop, study and provide metrological assurance for new sensitive methods for ^222^Rn measurements suited for low radon levels^4^ including these in the outdoor air^5^.

One approach to achieve high sensitivity to radon employs “concentrating” radon on proper collectors (adsorbents or absorbents) and measuring radiation from the collected radon and its progeny^6^. Probably, the most efficient “collector” of radon used so far is activated carbon. The ability of charcoal to adsorb radon was discovered by Sir Ernest Rutherford^7^. Nowadays activated carbon materials are widely used for ^222^Rn sampling and measurement^6,8–12^. The radiation from the adsorbed activity may be detected after^8,9^ or during exposure. In the second case the adsorbent is coupled with a detector (e.g. solid state nuclear track detectors (SSNTDs) that record tracks of alpha particles emitted from the adsorbed radon and its progeny)^6,10–12^. Recently, activated carbon fabrics were also used as effective “collectors/radiators”, particularly useful with SSNTDs, coupled with them^13,14^. Overall, the methods that employ activated carbon are highly sensitive, however they face serious limitations that hamper their application for long-term measurements or such in the outdoor environment. One limitation is the strong temperature dependence of the adsorption capacity. The adsorption capacity *k* (*k* = equilibrium ratio of specific activity of radon adsorbed on the adsorbent to the ambient ^222^Rn activity concentration) of activated carbon decreases dramatically with the increase of the temperature- e. g. by more than 200% over 8.5–31 °C^9^. Another limitation, that particularly hampers the potential for long term exposure is the adsorption of water, which leads to substantial loss of adsorption ability, when the occlusion of the pores by water happens^15^. So far the activated carbon-based methods are used mostly for short-term exposures—rarely for more than few days, and usually the activity is measured after the exposure, e. g. by liquid scintillation counting^9^.

A novel design of detector modules with compensated temperature dependence has been recently patented^16^. It employs placing detectors which sensitivity decreases with the increase of the temperature, like these based on activated carbon, in hermetic volumes, one or more walls of which are made of plastic material through which radon may diffuse. The fraction of radon penetrated by diffusion inside the volume increases with the increase of the temperature^17–19^. The key concept is to design proper “compensation modules”, in which these two independent and reciprocal trends compensate each other. In this work detector modules with radon detectors made by activated carbon fabrics coupled with SSNTDs are studied theoretically and experimentally. By computer modeling it is shown that compensation modules with sufficiently compensated temperature dependence might be designed. Experimental tests showed that the variation in the response to radon over 5–35 °C range can be reduced from 230–305% to 6–17%. In the same time the studied detector modules provide 7–9 times better sensitivity than conventional passive radon detectors. The compensation modules may be made thin, flexible and they could be useful for exposure under various environmental conditions. Such modules retard water penetration to the sensor inside, and protect it from thoron (^220^Rn, half-life 55.6 s) interference on the radon signal. These qualities of the module make possible to build new generation of radon detectors and monitors of much enhanced sensitivity and usable for long-term exposures at highly variable conditions that are typical for the atmospheric environment.

## Materials and methods

### Concept

**Figure 1:**
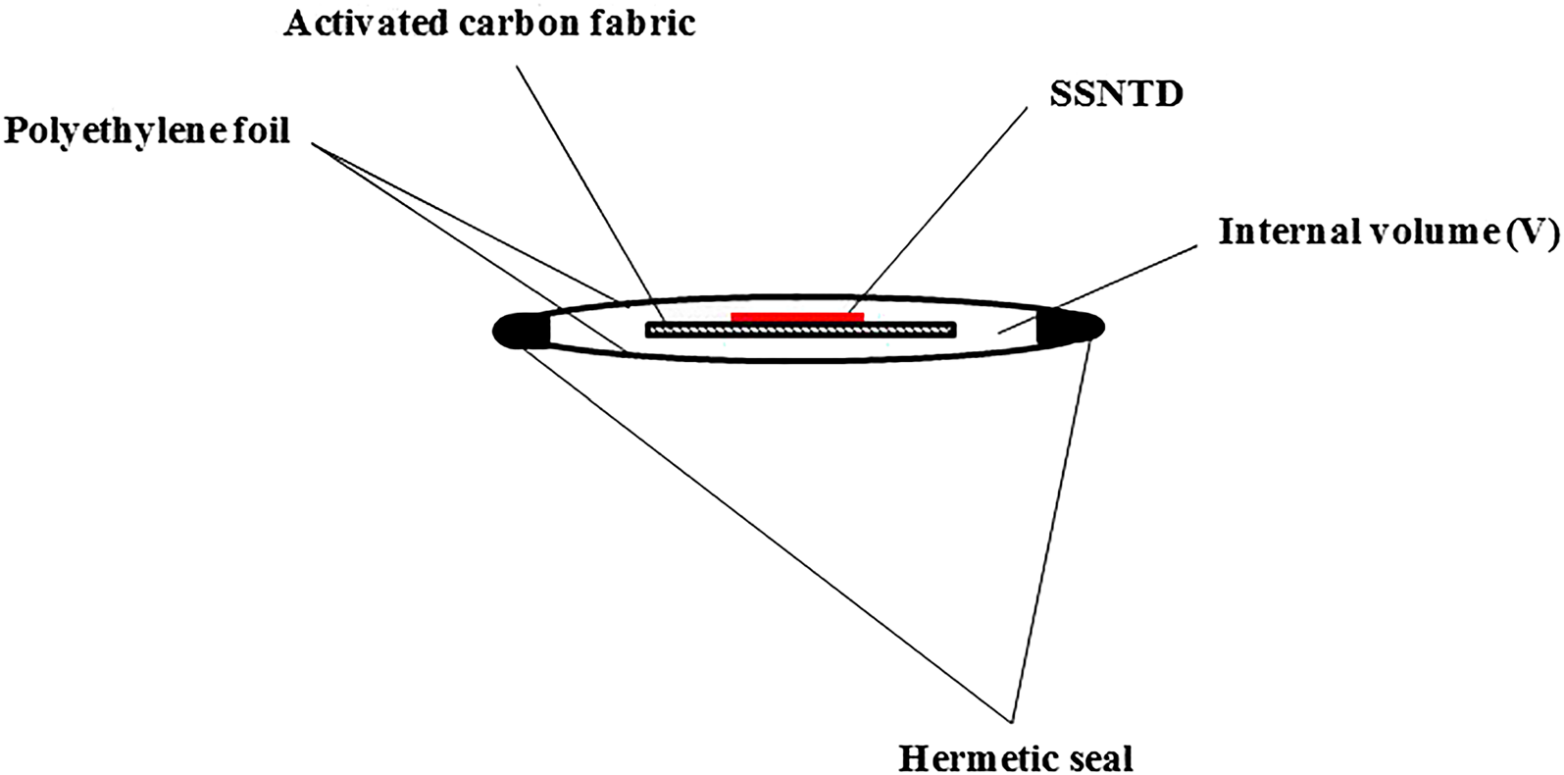
A scheme of the “compensation module” with a detector of activated carbon fabric and SSNTD of Kodak Pathe LR-115/II, used in the present experiments. The module is formed as a polyethylene envelope that wraps the detector of activated carbon fabric and the SSNTD. The hermetic sealing is ensured by heat sealing and silicon sealant.

The design of the detector module with temperature compensation is shown in Fig. 1. The sensor inside uses an adsorber of activated carbon fabrics coupled with SSNTD of Kodak-Pathe LR-115/II. In the present experiments three kinds of activated carbon fabrics (produced by Kynol Europa GmbH, Germany) were used: Kynol 507-10, ACC-10 and ACC-20. The activated carbon fabric serves as radon collector/radiator, and the SSNTD looks to it. The walls of the compensation module are made of foils of low density polyethylene (LDPE) or high density polyethylene (HDPE). The thickness and surface area of the foil is selected according to the modeling, aiming at achieving the best temperature compensation. The sensitivity of the detector is estimated by its calibration factor *CF* (*CF* = net-track density/integrated ^222^Rn activity concentration in the ambient air; the net-track density is the number of tracks per unit area, after the background is subtracted). When the detector is outside the detector module, the calibration factor is *CF*_*0*_. However, when the detector is placed inside its calibration factor, at equilibrium, will be *CF* = *CF*_*0*_*.ρ*, where *ρ* is the “penetration ratio”, the ratio at equilibrium of radon concentration in the air inside the module volume to the concentration outside it—in the ambient air. The *CF*_*0*_ is proportional to the adsorption capacity *k* and follows the same temperature trend as *k*, while *CF* is proportional to *kρ*. As noted elsewhere^17–19^, the fraction *ρ* that penetrates through the walls in the volume increases with the increase in the temperature—thus the trend with the temperature is reciprocal to that of *k*. The wall material through which the gas diffuses, its thickness, the wall surface area, and the volume of the “compensation module” are selected so that the temperature variation of *kρ* is at the possible minimum within the temperature interval of the applications.

### Modeling

The activated carbon fabrics used are sheets of thickness of few tenths of millimeter, so the process of adsorption may be expected to be fast. Using the model described elsewhere^20^, the evaluation of the time needed the adsorbed radon to reach equilibrium in the activated carbon fabrics was found to be of the order of few minutes, which is negligible compared to typical exposure durations. Further in the mathematical analysis, it is assumed that radon adsorption on the activated carbon fabric sheet occurs immediately, without inertia.

Here, the theory presented in Ref.^19^ is modified to consider the adsorption on an activated carbon fabric element of mass *m* placed in the compensation module volume. A closed volume *V* (Fig. 1) with a surface area *S* of the sides through which the radon diffuses is considered. The thickness of the polymer foil of which the sides are made is *h*, the diffusion coefficient of radon in the polymer material is *D*. The radon diffusion through the foil is described by the equation:

1 $$\frac{\partial c}{{\partial t}} = D\frac{{\partial^{2} c}}{{\partial x^{2} }} - \lambda c,$$

where *c* is the radon activity concentration in the polymer material and *λ* is the decay constant of ^222^Rn. Equation (1) is considered with the initial condition *c*(*t* = *0, x*) = 0 and boundary conditions *c*(*t, x* = *0*) = *Kc*_*out*_(*t*) and *c*(*t, x* = *h*) = *Kc*_*in*_(*t*), where *x* = *0* is the coordinate of the external surface of the foil, *x* = *h* is that of its internal surface. The quantities *c*_*out*_(*t*) and *c*_*in*_(*t*) are the ^222^Rn activity concentrations in the air outside and inside the volume, respectively, and *K* is the partition coefficient of the foil material (the partition coefficient is the dimensionless solubility of the material that is the ratio, on the border, of the concentration in the material to that in the air). Inside the volume the activity is distributed between air and the activated carbon sheet. Assuming fast distribution, without inertia in adsorption process, the total activity inside is^20^:

2 $$A_{in} = \, c_{in} V + c_{in} km \, = \, c_{in} \left( {V + km} \right) \, = \, c_{in} V^{\prime},$$

where the “equivalent volume” *V’* is defined as *V’* = *V* + *km*.

The time dependence of the concentration inside the chamber volume can be modeled by the equation:

3 $$\frac{{dA_{in} }}{dt} = \frac{\text{d}c_{in}}{\text{d}t}V^{\prime}=\left. { - SD\frac{\partial c}{{\partial x}}} \right|_{x = h} - \lambda A_{in} = \left. { - SD\frac{\partial c}{{\partial x}}} \right|_{x = h} - \lambda c_{in} V^{\prime}.$$

The first term in the right-side of Eq. (3) describes the flux by Fick’s diffusion from the internal surface of the foil, while the second term is for the radioactive decay. In this report integrated mode of measurement is considered so the net track density is proportional to the integrated radon concentration for a given exposure time *t*_*exp*_. The integrated activity concentrations will be denoted by capital symbols, e.g.:

4 $$C = \mathop \smallint \limits_{0}^{{t_{exp} }} c\left( t \right)dt,$$

The equations and solutions in present case are equivalent to these described and solved elsewhere^19^, when replacing in them *V* with *V′* = *V* + *km*.

The expression for *ρ*, according to the corresponding expression (Eq. (16) in^19^), will be:

5 $$\rho = \frac{{C_{in} }}{{C_{out} }} = \frac{1}{{1 + \lambda \frac{hV^{\prime}}{{PS}}}} = \frac{1}{{1 + \lambda \frac{{h\left( {V + km} \right)}}{PS}}},$$

where *P* = *KD* is the permeability of the polymer material of walls for radon. As the *CF* is proportional to *kρ*, the approach of the module design is to choose, for a given *m*, a polymer material and values of *h*, *V* and *S* of the foil used, so the expression:

6 $$\eta = k\rho = \frac{k}{{1 + \lambda \frac{{h\left( {V + km} \right)}}{PS}}},$$

to be constant, or to vary minimally with the variation of the temperature. Notably, when *V* << *km* (which was the case in present designs), the influence of *V* becomes negligible and the compensation, for a given *m* and polymer material, remains to depend only on *h* and *S*.

The temperature dependence of the adsorption capacity can be expressed as^8,21^:

7 $$k\left( T \right) = \kappa \times exp\left( {\frac{Q}{{R\left( {T + 273.16} \right)}}} \right),$$

where R = 8.31441 J mol^−1^ K^−1^ is the molar gas constant, *T* is the temperature (°C), *Q* is the heat of adsorption of radon on activated carbon (23 999 J mol^−1^, according to the value given in^9^). At this step, for the purposes of modeling we used for *k* also data given in the literature, assuming *k* = 4 m^3^ kg^−1^ at 21 °C (according to^20^, where this value is recommended as a typical value at room temperature). Thus, the corresponding value of the constant κ in Eq. (7) will be *κ* = 2.19 × 10^–4^ m^3^ kg^−1^. The values of *ρ* were modeled using data for radon permeability and penetration through LDPE and HDPE, described in^19^ and the algorithm, described there. This way $\eta \left( T \right) \, = \, k\left( T \right)\rho \left( T \right)$ was calculated for temperatures within the interval 5–35 °C.

### Experimental

Experiments were made at 3 different temperatures (kept constant within ± 0.5 °C): 5 °C, 21 °C and 35 °C using the laboratory exposure facility described elsewhere^22^. The exposures were made at integrated ^222^Rn concentrations of 56.2 ± 3.9 kBq h m^−3^, 59.9 ± 4.2 kBq h m^−3^ and 58.8 ± 4.1 kBq h m^−3^, respectively. During exposure the activity concentrations of ^222^Rn were followed by a reference radon monitor AlphaGUARD PQ 2000 PRO Rn Tn (Genitron GmbH). Pairs of one packed in the module and one non-packed detector with each kind of activated carbon fabrics used, were loaded in the exposure chamber. Another, “background” set of the same pairs was left outside the chamber—at low radon levels (about 20 Bq m^−3^) but at the same temperature as in the exposure chamber. After exposure all exposed sets were left for one week to degas at low radon levels (20 Bq m^−3^) at the same temperatures at which the exposure was made. After out-gazing, the sensors were dismantled and the LR-115/II detectors were removed and etched at 10% NaOH for 100 min at 60.0 ± 0.1 °C. After etching they were washed for 30 min in flushing water and left for 2 min in 50% ethanol. After that they were left to dry and the tracks were counted visually by a microscope to obtain the track-density. The track-density of the “background” detectors, which is due to the detector background and the low radon exposure during detectors storage for out-gasing, was subtracted from the track density of exposed detectors to obtain the “net track-density”.

## Results

### Compensation module design

**Figure 2:**
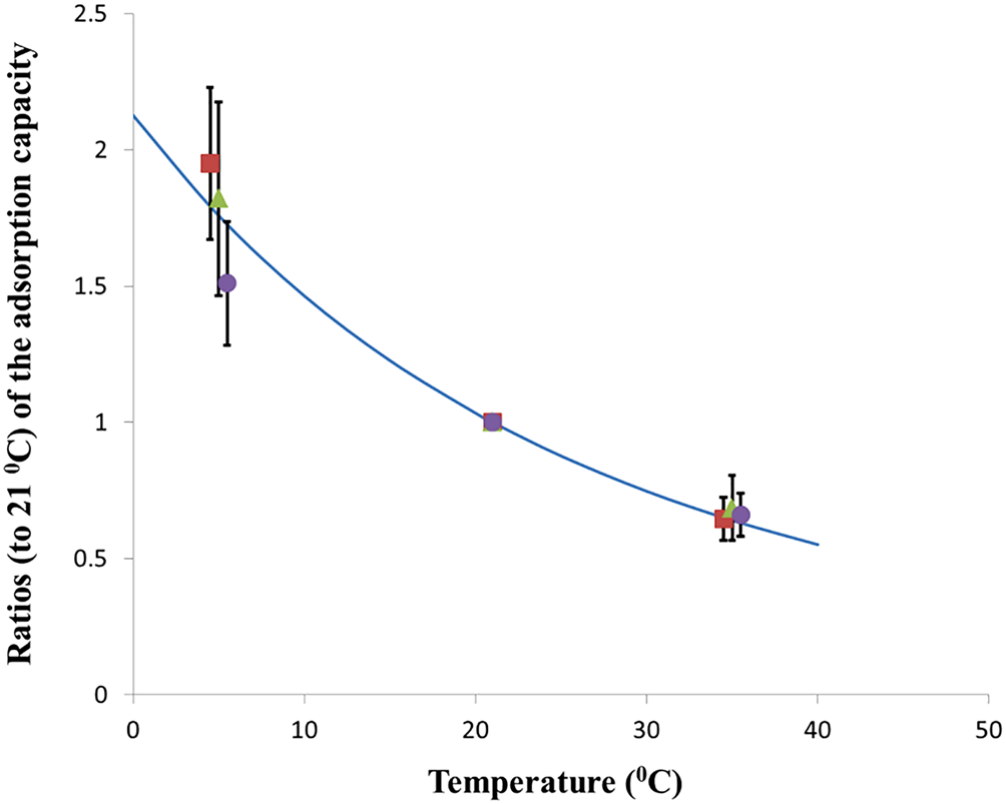
The temperature dependence of the adsorption capacity, illustrated by the experimental ratio $CF_{0} \left( T \right)/{\text{CF}}_{0}$ (21 °C) (points) and theoretical $k\left( T \right)/k$ (21 °C) (the curve), where k-values are calculated using Eq. (7). The experimental points are related to detectors with Kynol 507-10 (), ACC-10 () and ACC-20 (). The points at 5 and 35 °C are slightly deflected horizontally, for better visibility.

To verify the assumed temperature trend of *k* (the temperature trend in *CF*_*0*_ is the same) the experimental results of detectors exposed outside the modules were used. The results are illustrated in Fig. 2. As seen the experimental results fit well the theoretical curve.

**Figure 3:**
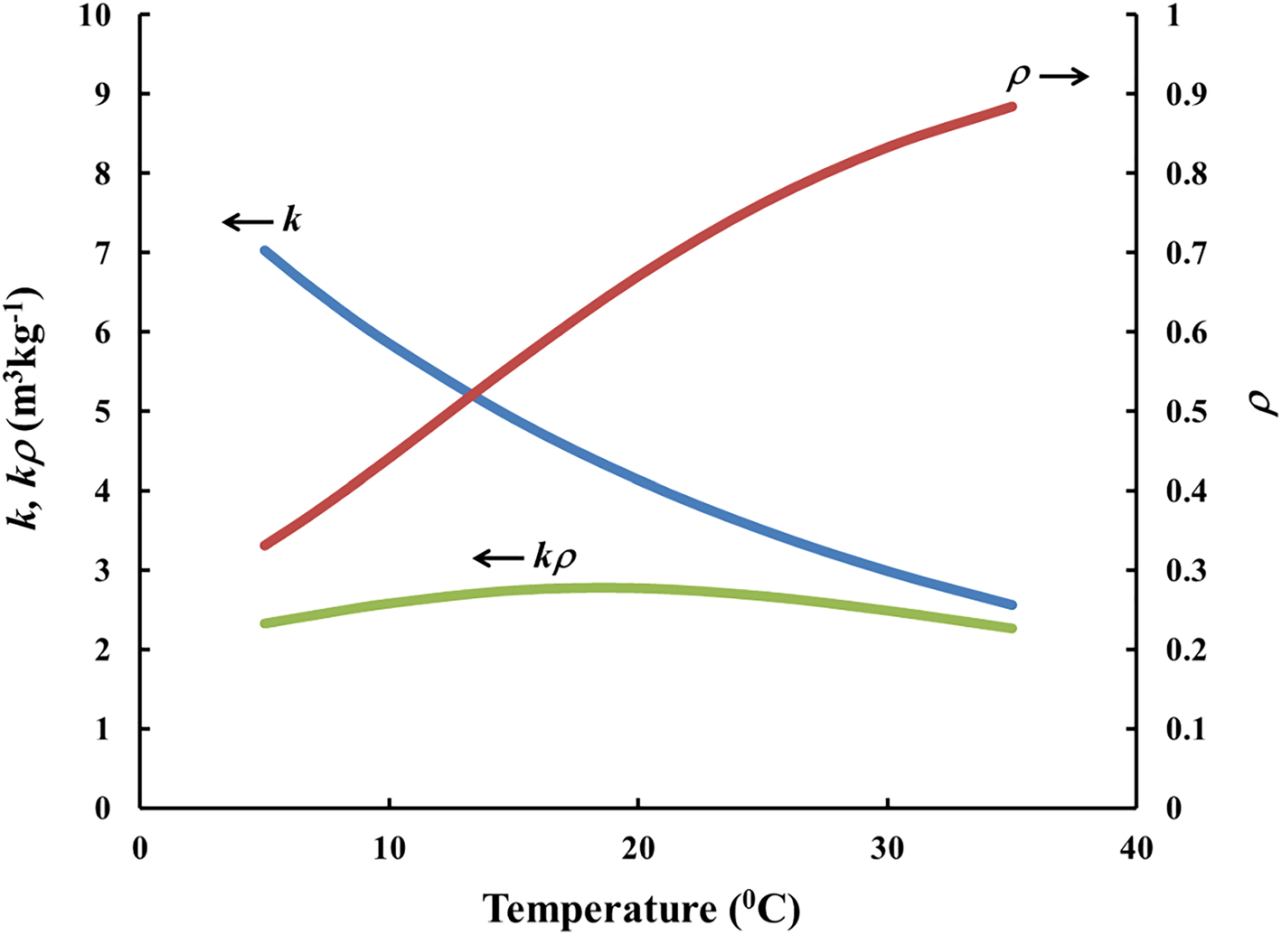
Theoretical modeling of the temperature compensation achieved with a module of *S* = 290 cm^2^ made of HDPE foil of thickness 9 μm. The mass of the adsorbent inside is assumed *m* = 500 mg.

The modelling used to design the individual compensation modules can be illustrated on the example of adsorber of Kynol 507-10 fabric of mass *m* = 500 mg and a polymer foil of 9 µm thick HDPE foil. As the real compensation modules used in the experiments were wrapped to the sensor so that *V* <  < *km*, the same was assumed for the considered module (for the assumed mass of 500 mg km = 2000 cm^3^ at 21 °C). Criterion for the optimum design was based on the minimum of the relative “standard deviation” (*RSD*) of *η* over the temperature range of interest (*T*_*1*_, *T*_*2*_), given by the expression:

8 $$RSD = \frac{1}{{\overline{\eta }}}\sqrt {\frac{1}{{T_{2} - T_{1} }}\mathop \int \limits_{{T_{1} }}^{{T_{2} }} \left( {\eta - \overline{\eta }} \right)^{2} dT} ,$$

where $\overline{\eta }$ is the average value of *η* = *kρ* over (*T*_*1*_, *T*_*2*_) . In the present research the temperature range of interest was 5–35 °C. In such case, the best compensation, according to the “minimum RSD” criterion was reached when *S* = 290 cm^2^, e. g. each of the sides of the envelope (“the compensation module”) is of area of 145 cm^2^, which corresponds e. g. to a square of side of about 12 cm (more precisely: 12.04 cm). Figure 3 illustrates the results. According to the model the *RSD* of *kρ*, and therefore that of *CF*, over 5–35 °C range may be reduced to 7.2% (the ratio maximum/minimum for *kρ* in the temperature range specified is 1.22, while that of *k* is 2.75, i. e. the value of *k* at 5 °C is 275% that at 35 °C).

### Experimental results

For experiments individual modules were designed for detectors using fabrics of Kynol 507-10, ACC-10 and ACC-20. For Kynol 507-10 and ACC-20 the modules were of HDPE and for ACC-10 of LDPE. The individual design followed the methodology described above, taking into account the exact individual mass of each fabric. In all designs the value of *k(T)* at different temperatures was calculated according to Eq. (7).

**Figure 4:**
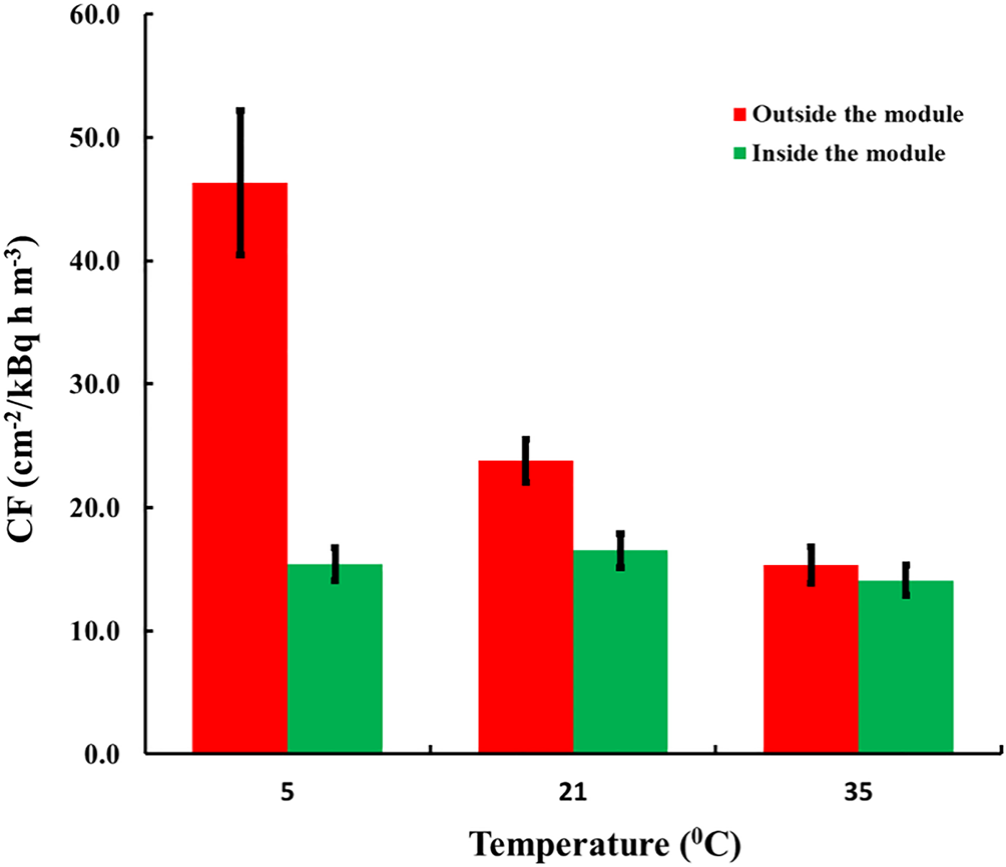
Experimental results with adsorbers of Kynol 507-10 exposed outside (*CF*_*0*_) and inside (*CF*) modules of 9 µm thick foil of HDPE.

The results for Kynol 507-10 are illustrated in Fig. 4. The *CF*_*0*_ at 5 °C is by 305% higher than *CF*_*0*_ at 35 °C (maximum/minimum = 3.05). In contrast, for the sensors placed in the compensation module the variation of *CF* is within 17% (max/min = 1.17). Notably, the paired differences between the *CFs* of the sensors exposed at different temperatures inside the modules were not statistically significant at 95% level of significance.

**Figure 5:**
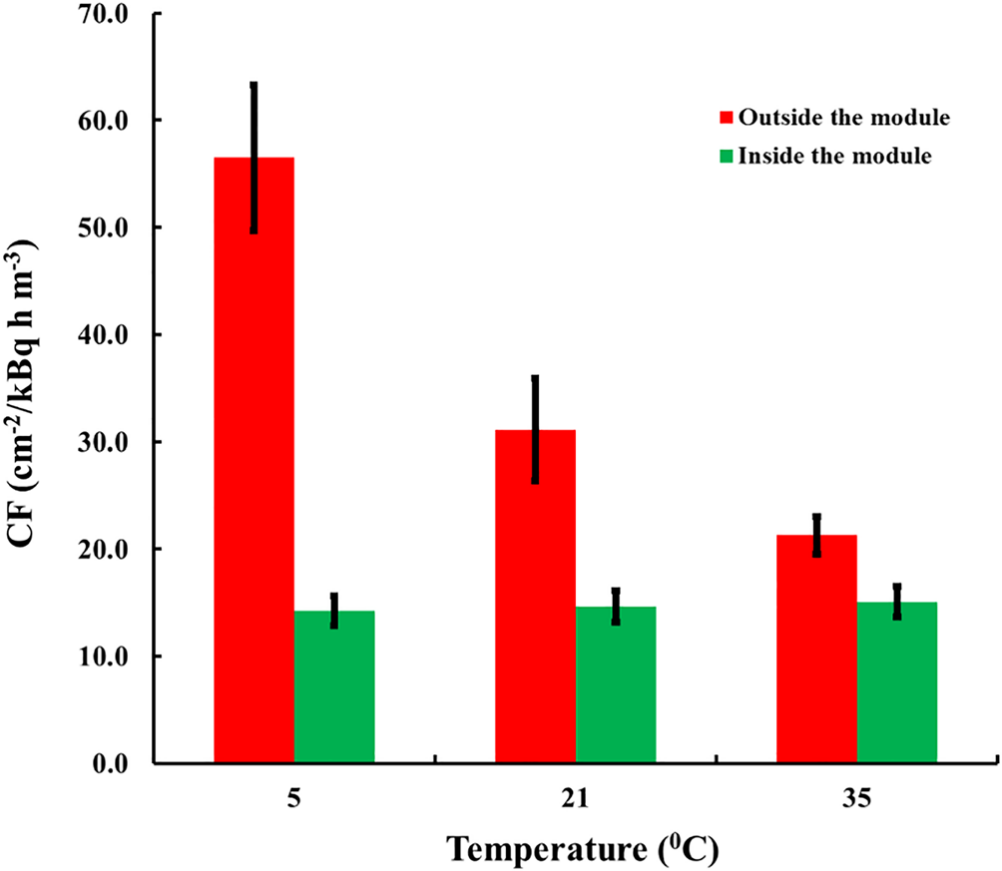
Experimental results with adsorbers of ACC-10 fabric of sensors exposed outside (*CF*_*0*_) and inside (*CF*) modules of 20 µm LDPE foil.

The results of ACC-10 were obtained with modules of LDPE and are illustrated in Fig. 5. Outside the module, the variation of *CF*_*0*_ is 265% (max/min = 2.65). In modules, the variation of *CF* is within 6% (max/min = 1.06), and the paired differences were not statistically significant.

**Figure 6:**
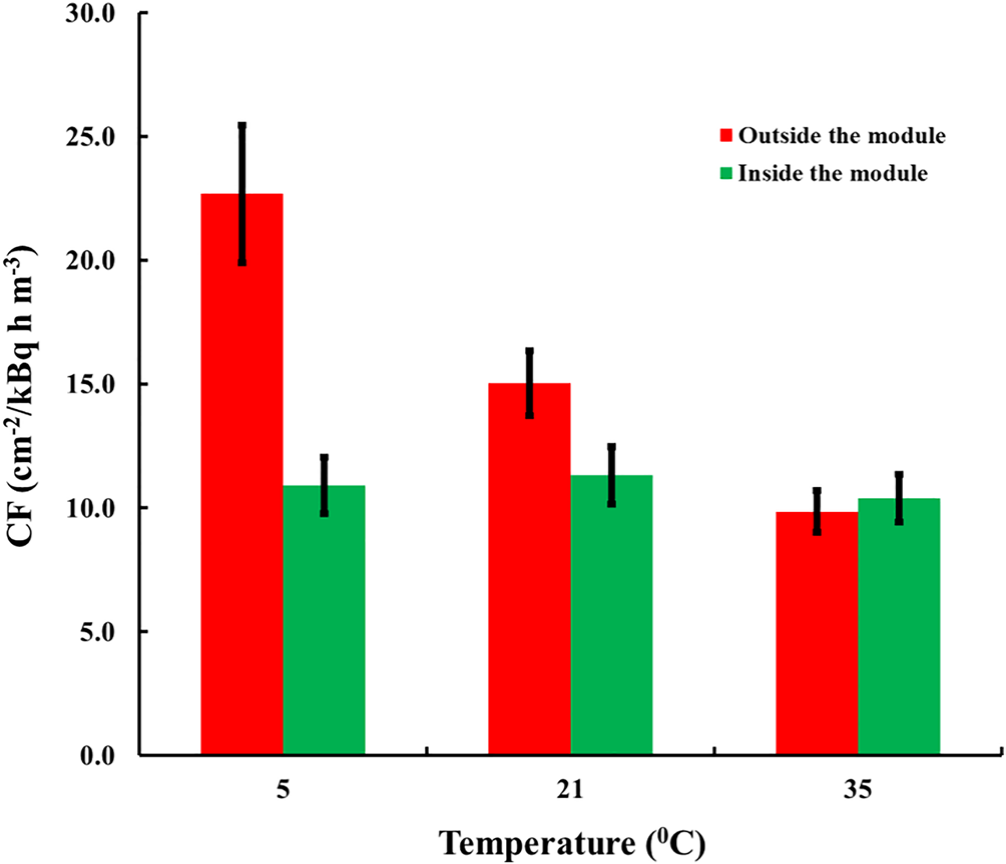
Experimental results with adsorbers of ACC-20 fabric of sensors outside (*CF*_*0*_) and inside (*CF*) modules of 9 µm HDPE foil.

The experiment with ACC-20 was with modules of 9 µm HDPE and the results are illustrated in Fig. 6. In this case for *CF*_*0*_ the variation is 230% (max/min = 2.3). Again, inside the module the temperature dependence is practically fully compensated, the variation being within 9% (max/min = 1.09) and the paired differences were not statistically significant.

The *CFs* of described detectors with compensation modules are significantly higher than these of other commonly used passive radon detectors. The average *CFs* with different fabrics for the studied compensation modules are as follows:

- Kynol 507-10: 15.4 ± 1.3 cm^−2^/kBq h m^−3^;
- ACC-10: 14.7 ± 0.9 cm^−2^/kBq h m^−3^;
- ACC-20: 10.9 ± 0.8 cm^−2^/kBq h m^−3^;

For comparison, for passive radon detectors (diffusion chambers) with SSNTDs of CR-39 *CF* of 2.09 ± 0.21 cm^−2^/kBq h m^−3^ is reported^23^. The diffusion chambers^19^ that use the same sort of track detectors (LR-115/II) as described in the present work, and etched at the same etching conditions are of *CF* = 1.65 cm^−2^/kBq h m^−3^. As seen, detector modules with Kynol 507-10 or ACC-10 provide 7–9 times better sensitivity than that of commonly used passive radon detectors.

**Figure 7:**
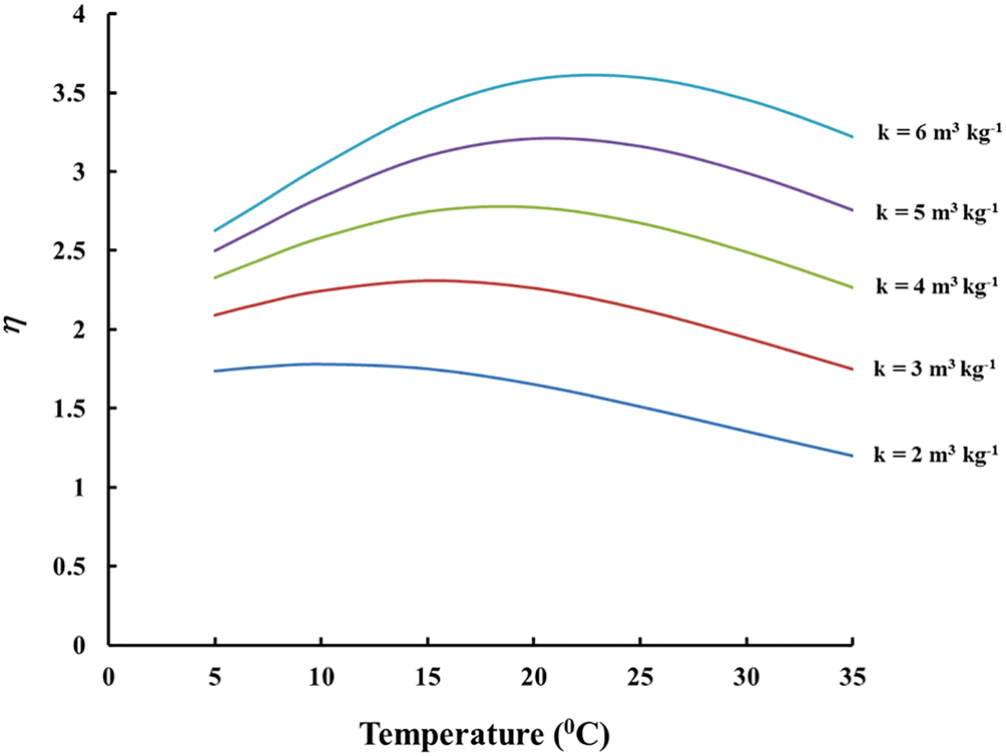
Temperature dependence of *η* = *kρ* of the module of Fig. 3 for different values of *k*.

Looking at the results for detectors exposed outside the modules (Figs. 4, 5, 6) it can be seen that *CF*_*0*_ at one and the same temperature differs between fabrics. This implies some difference in the adsorption capacity of the different fabrics. One may provisionally rank the adsorption capacity as ACC-10 ≥ Kynol 507–10 > ACC-20. In all modules design the value of the adsorption capacity at 21 °C of 4 m^3^ kg^−1^ was assumed. However as our experimental results as well as the literature data suggest possible variances of *k* of different activated carbon materials. The activated carbon data in the literature show variations in the range 2–6 m^3^ kg^−1^ for adsorption capacity at room temperature^20^. To clarify the possible influence of the uncertain data for *k* on the quality of module designs, computer simulations with different *k*-values were made. The results can be illustrated on the example of the HDPE compensating module modelled in Fig. 3. The results for different *k*-values are illustrated in Fig. 7. As seen, for all values of *k* within the interval 2–6 m^3^ kg^−1^, the variation of *η* (e. g. the ratio max/min) with the temperature remains much less than the variation of 230–305% observed with the unpacked sensors.

**Figure 8:**
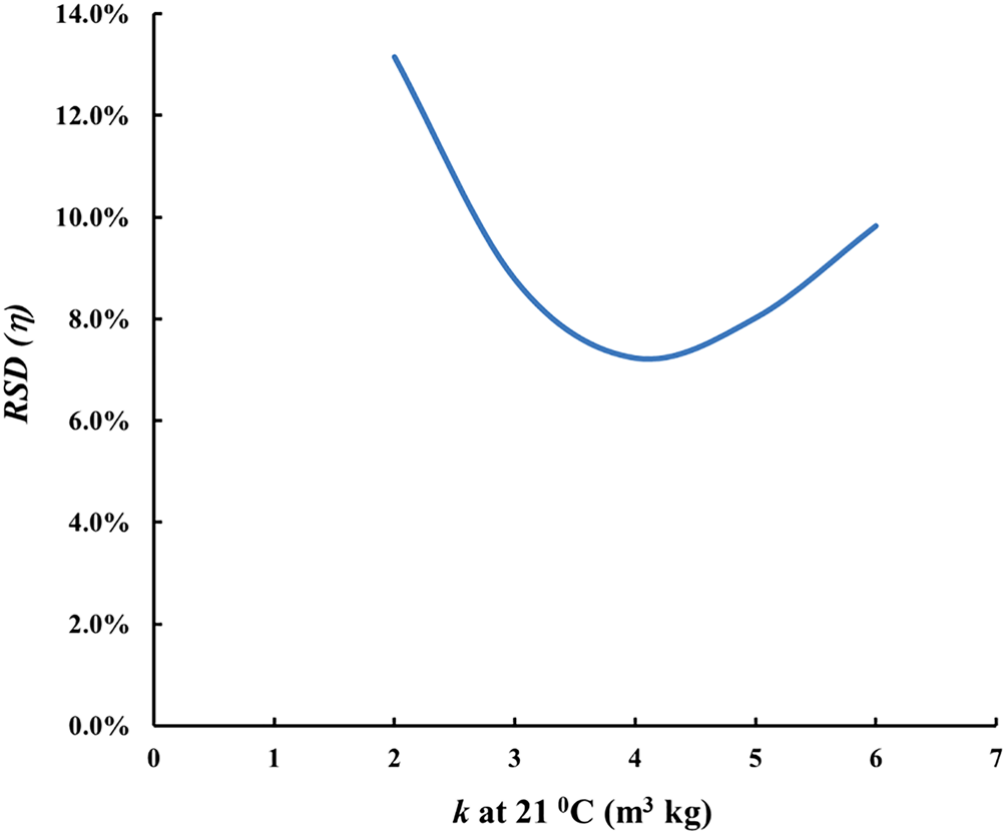
Relative standard deviation in *η* over 5–35 °C as dependent on *k* for the module from Fig. 3.

This can be seen clearly in Fig. 8, which illustrates the *RSD (η)* in the temperature range 5–35 °C, as dependent on *k*. At *k* = 4 m^3^ kg^−1^ it is 7.2%, and at variations of *k* in the indicated interval of *k*-values *RSD* does not exceed 13.2%. It can be concluded that the influence of inaccuracies in *k*-values used for the design of the compensating module on the quality of the temperature compensation is relatively limited.

## Discussion and conclusions

A novel highly sensitive passive radon detectors based on activated carbon fabrics with compensation of the temperature influence is proposed. The detectors studied in the present work are made of activated carbon fabric + SSNTD. The detector is placed in a “compensation module”^16^ that reduces significantly the temperature influence on the adsorption capacity of the adsorbent used. Modelling revealed that with such design the temperature variation (max/min) of *CF* within 5–35 °C range may be reduced from 230–305% to 6–17%, according to the experimental results.

Such radon detectors may be made flat, very compact and flexible. They demonstrate substantially higher *CF* than that of other conventional radon detectors^19,23^ and this may pave new roads for application. For instance, the detection limit for 6 months exposure, for the radon detectors reported in^23^ is 4 Bq m^−3^. The sensitivity of other passive radon detectors is comparable to this estimate (e.g. 30 Bq m^−3^ for one month of exposure, which is 5 Bq m^−3^ for 6 months as summarized in^1^). With 7–9 time better sensitivity, the design presented here has potential to achieve detection limit of < 1 Bq m^−3^ for six months of exposure and few Bq m^−3^ for one month. The sensitivity might be increased even more by using large area detectors of low background, as those described in^24^. Future research will be focused also on the possibility to use active detectors (e. g. semiconductor detectors of alpha particles) with the compensation modules described.

One more benefit of these modules is that the loss of adsorption capacity due to the humidity is also expected to be greatly retarded, as polymer foils provide efficient, although not absolute, protection against moisture^25^. So far the humidity influence is the factor that limits the exposure duration of activated carbon based detectors to about one week maximum, and even less than one day at high humidity levels^8^.

Nowadays, radon detectors are required to have low interference of thoron on the signal^26^. The novel design provides also efficient protection against thoron (^220^Rn) interference on the radon signal: Because of its short half-life (55.6 s) thoron almost fully decays during diffusion through the polymer foil of the walls of the module, which is not the case for radon. Using Eq. (5) with the decay constant of thoron, it was calculated that, for the module illustrated in Fig. 3 and for temperatures in the range 5–35 °C, the penetration ratio for thoron is 0.007–0.15%, while for radon it is 30–90% (see Fig. 3).

These qualities make possible the design of a new generation of highly sensitive radon detectors and monitors, applicable for short as well as for long-term measurements under wide range of environmental and temperature conditions. Novel designs of sensitive radon detectors are explored as it is planned to include such monitors in climate monitoring networks, including the European Integrated Carbon Observation System (ICOS). The detectors proposed in the present work may be suitable for that purpose. This however needs extensive dedicated research on the detector background, the possibility to use large-area detectors of very low intrinsic background^24^ and the humidity resistance of different compensation modules. Different kinds of activated carbon materials and, possibly, other adsorbents should be studied to find out the most efficient radon adsorbent. It should be noted, that so far the design is based on crude data for *k* and radon permeability of HDPE and LDPE. More detailed experimental studies of these quantities at different temperatures and for different adsorbents and plastics are planned.

One may speculate on the possibilities to use similar “compensation modules” for detection of the environmental radioxenon (mostly ^133^Xe). Radioxenon attracts substantial attention in the light of current challenges in nuclear security and Treaty on the Non-Proliferation of Nuclear Weapons and the Comprehensive Test Ban Treaty^27^. In this case the module should be coupled with a proper beta/gamma radiation detector. Progress in this direction would need dedicated research on the adsorption capacity of different adsorbents for xenon and its transport properties in different polymer materials.

The proposed detectors with compensation modules provide highly sensitive option for monitoring radon and, possibly radioxenon, too in the environment. However, practical application of such “compensation modules” may need some more data on adsorption properties of the prospective adsorbents at different temperatures, as well as on solubility and diffusion of radon and, possibly, other radioactive noble gases too in different polymer materials.

## Data Availability

The datasets generated and/or analyzed during the current study are available in the Research Gate repository: https://www.researchgate.net/publication/358977684_Data_used_on_individual_modules_design_optimization_of_walls_area.
